# Transmission of *Bordetella holmesii* during Pertussis Outbreak, Japan

**DOI:** 10.3201/eid1807.120130

**Published:** 2012-07

**Authors:** Hajime Kamiya, Nao Otsuka, Yuka Ando, Fumito Odaira, Shuji Yoshino, Kimiko Kawano, Hirokazu Takahashi, Toshihide Nishida, Yoshio Hidaka, Hiromi Toyoizumi-Ajisaka, Keigo Shibayama, Kazunari Kamachi, Tomimasa Sunagawa, Kiyosu Taniguchi, Nobuhiko Okabe

**Affiliations:** National Institute of Infectious Diseases, Tokyo, Japan (H. Kamiya, N. Otsuka, Y. Ando, F. Odaira, H. Toyoizumi-Ajisaka, K. Shibayama, K. Kamachi, T. Sunagawa, K. Taniguchi, N. Okabe);; Miyazaki Prefectural Institute for Public Health and Environment, Miyazaki, Japan (S. Yoshino, K. Kawano);; Takahashi Clinic, Miyazaki (H. Takahashi);; and Nobeoka Public Health Center, Miyazaki (T. Nishida, Y. Hidaka)

**Keywords:** Bordetella infections, pathogen transmission, outbreak, Bordetella holmesii, Bordetella pertussis, pertussis, bacteria, Japan

## Abstract

We describe the epidemiology of a pertussis outbreak in Japan in 2010–2011 and *Bordetella holmesii* transmission. Six patients were infected; 4 patients were students and a teacher at the same junior high school. Epidemiologic links were found between 5 patients. *B. holmesii* may have been transmitted from person to person.

*Bordetella holmesii*, a small gram-negative coccoid bacillus that was first reported in 1995 (*1*), was originally identified as a member of the Centers for Disease Control and Prevention nonoxidizer group 2. The organism is associated with bacteremia, endocarditis, and pneumonia, usually in patients with underlying disorders such as asplenia or sickle cell anemia, and has been isolated from blood and sputum samples (*1**–**5*). *B. holmesii* may also be responsible for causing symptoms similar to those of pertussis (whooping cough) in otherwise healthy patients (*6*). Large surveillance studies in the United States and Canada have shown that the organism was detected in nasopharyngeal swab (NPS) specimens of patients with pertussis-like symptoms (*7**,**8*). Although humans may be infected with *B. holmesii*, transmission of *B. holmesii* between humans has not yet been fully elucidated.

Pertussis is a highly contagious disease caused by the bacterium *B. pertussis*. The organism is known to be transmitted from person to person by airborne droplets (*9*). During a recent pertussis outbreak, we conducted a laboratory-based active surveillance study and detected 76 suspected cases of pertussis. Among these cases, we identified not only *B. pertussis* infection but also *B. holmesii* infection. The purpose of this study was to describe the epidemiology of the pertussis outbreak and to determine the epidemiologic relatedness of *B. holmesii* transmission.

## The Study

During 2010–2011, a pertussis outbreak occurred in a suburban town (town A) in Nobeoka City in Miyazaki Prefecture, Japan. Town A has a population of 4,227 persons and an elementary school and junior high school. The first pertussis case (in a 17-year-old boy) was reported in September 2010. From that time, we conducted a laboratory-based active surveillance study in the area until April 2011, in cooperation with clinics, hospitals, and local health departments. Pertussis-suspected cases were defined as an illness with cough lasting for >2 weeks, and pertussis-confirmed cases were defined as the presence of 1 of the following laboratory findings: a culture-positive result for *Bordetella* species from NPS specimens, or a positive result for molecular testing for *Bordetella* species.

For molecular testing, we conducted conventional PCR specific for insertion sequence IS*481*, which is known to detect *B. pertussis* and *B. holmesii*, and *B. pertussis*–specific loop-mediated isothermal amplification assays (*10**–**12*). To further confirm *B. holmesii* infection, *B. holmesii*–specific real-time PCR specific for the *recA* gene was also performed as described (*8*). For confirmed cases of *B. holmesii* infection, we collected the general information for patients (clinical symptoms, treatment, contact information, and outcome) by face-to-face interview or questionnaire.

**Figure 1:**
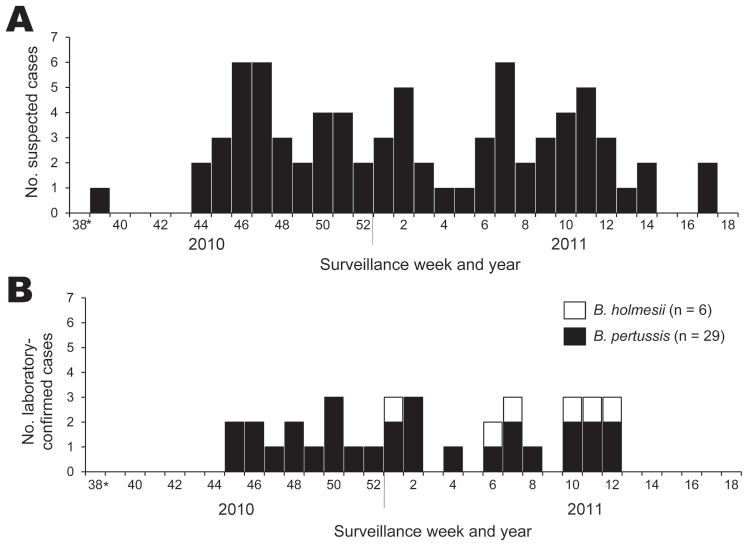
Epidemic curve of a pertussis outbreak, September 2010–April 2011, Japan. A) Suspected cases of pertussis. B) Laboratory-confirmed cases of *Bordetella pertussis* and *B. holmesii* infection. *As of September 20–26, 2010.

During the pertussis outbreak, we identified 76 suspected pertussis cases. Among these suspected cases, 35 cases were confirmed by laboratory testing and involved persons 2–63 years of age (median age 13 years); 1 case occurred in a 2-year-old child, 14 in 6- to 12-year-old children, 14 in 13–15-year-old children, and 6 in persons >16 years of age. Despite pertussis vaccination rates in childhood of 82.3%−92.6%, most (80%) patients were students 6–12 and 13–15 years of age. Among the 35 confirmed case-patients, 29 and 6 patients showed *B. pertussis* and *B. holmesii* infection, respectively. Confirmed cases of *B. holmesii* infection were observed within the last half of the epidemic curve, i.e., weeks 1–12 of 2011 (Figure 1). There were no cases of co-infection with *B. pertussis* and *B. holmesii*.

All NPS specimens from patients with *B. holmesii* infection showed a negative result for the *B. pertussis* loop-mediated isothermal amplification, but showed positive results in the IS*481* PCR and *B. holmesii recA* real-time PCR (Table 1). *B. holmesii*-like organisms were obtained from 5 patients, and these were identified as *B. holmesii* by *recA* gene sequencing. Patient 6 had been treated with antimicrobial drugs before the culture test, which probably resulted in a culture-negative test result in this patient. Real-time PCR confirmed that patient 6 had a low *B. holmesii* DNA load (threshold cycle 36.6) in her NPS specimen. All patients experienced paroxysms of coughing without posttussive vomiting, especially at night, and 3 also experienced a whoop (Table 2). Five of 6 patients had a persistent cough lasting >2 weeks. No patients experienced any complications, and they were treated mainly with azithromycin, resulting in complete recovery. None of the patients were immunocompromised.

**Table 1:** Characteristics of *Bordetella holmesii* infection in 6 patients during pertussis outbreak, Japan, September 2010–April 2011*

Patient no.	Age, y/sex	Duration of cough, d†	*B. holmesii* test results	
			*recA* real-time PCR (C_t_)‡	Culture§
1	17/M	5	+ (28.7)	+
2	15/F	4	+ (23.4)	+
3	15/F	>14	+ (21.6)	+
4	14/F	8	+ (25.1)	+
5	40/M	8	+ (27.0)	+
6	45/F	15	+ (36.6)	–

*All patients had negative results for *B.* *pertussis* loop-mediated isothermal amplification and positive results for IS*481* PCR. C_t_, cycle threshold; –, negative; +, positive. †At time of specimen collection. ‡Detection limit was a C_t_ value of 38.7, corresponding to 100 fg of DNA of *B. holmesii* ATCC51541. §All strains were isolated from nasopharyngeal swab specimens.

**Table 2:** Clinical and epidemiologic characteristics for 6 *Bordetella holmesii–*infected patients during pertussis outbreak, Japan, September 2010–April 2011*

Patient no.	Whoop	Duration of cough	Treatment	DTP vaccine status, no. doses	Medical history	Epidemiologic findings
1	+	10 d	AZM	4	Asthma	Student at high school A. His 14-year-old sister, who was given a diagnosis of pertussis, was a student at junior high school B.
2	+	28 d	AZM	4	−–	Student at junior high school B. Her 18-year-old brother had similar symptoms, but laboratory test results were negative.
3	–	>4 wk	AZM	4	–	Student at junior high school B. Her close friends began coughing after her disease onset.
4	+	15 d	AZM	4	Chlamydial pneumonia	Student at junior high school B. Her 11-year-old sister was given a diagnosis of pertussis before her disease onset.
5	−–	28 d	AZM	UNK	Allergic rhinitis	Teacher at junior high school B in charge of patient 4.
6	–	23 d	AZM, CFPN-PI, GRNX	UNK	Rheumatoid arthritis	Medical staff at clinic C, which was visited by patients 1–5.

*All patients had a paroxysmal cough and coughed at night; none had posttussive vomiting. DTP, diphtheria-tetanus-pertussis; +, positive; AZM, azithromycin; –, negative; UNK, unknown; CFPN-PI, cefcapene pivoxil; GRNX, garenoxacin.

**Figure 2:**
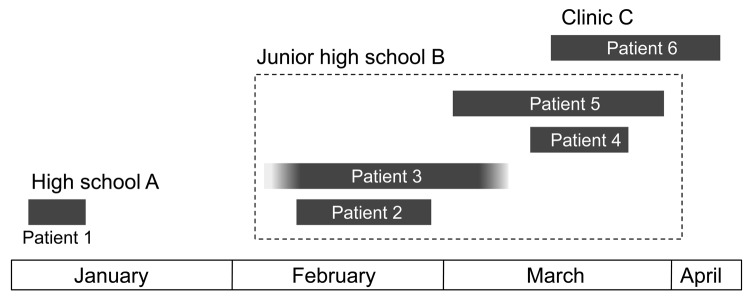
Epidemiologic linkage in 6 patients infected with *Bordetella holmesii* during pertussis outbreak, Japan, 2011. Duration of illness for each patient is shown as a gray box. Patient 3 provided unreliable information about the date of onset and recovery, but the patient’s cough lasted for >1 month. Epidemiologic linkage was observed between 5 patients (patients 2–6), but not for patient 1.

Our surveillance study showed epidemiologic linkage between 5 patients (patients 2–6), but not for patient 1 (Figure 2). Patient 1, a student in high school A, had the first case of *B. holmesii* infection (probable index case). He lived in a dormitory outside Nobeoka City. However, his family home was in town A in Nobeoka City, and he returned to his home at the end of 2010 and remained there in 2011. Patients 2, 3, and 4 were students at the same junior high school (B) in town A and were close friends. Patient 5 was a teacher at junior high school B and was in charge of patient 4. Patient 6 was a medical practitioner at clinic C, which is 1 of 2 clinics in town A. Patients 1–5 visited clinic C in early January, late February, late February, early March, and late March, 2011, respectively. The duration of illness in patient 1 did not overlap with that of the other patients, whereas that of patients 2–6 clearly overlapped.

## Conclusions

In the past 16 years, ≈70 *B. holmesii* clinical strains have been isolated from human patients in several countries, mainly the United States. All reported cases of *B. holmesii* infection have been sporadic occurrences. Thus, the reservoir of *B. holmesii* is currently unknown. Moreover, whether *B. holmesii* is transmitted between humans is not known. In this report, we have demonstrated that 5 patients infected with *B. holmesii* showed epidemiologic linkage. In particular, the fact that 4 of these patients attended the same junior high school suggests that *B. holmesii* may be transmitted from person to person.

A previous report suggested that *B. holmesii* and *B. pertussis* may co-circulate in young adults (*7*). However, the relationship between pertussis epidemics and *B. holmesii* infection is not fully understood. Our active surveillance study showed that *B. holmesii* infection spread concurrently with the *B. pertussis* epidemic, but that there was no co-infection of *B. holmesii* and *B. pertussis*. Our observations demonstrate that accurate diagnosis is needed to discriminate between *B. holmesii* and *B. pertussis* infections during a pertussis outbreak because symptoms associated with these 2 diseases are similar.

In 2012, a patient with *B. holmesii* infectious pericarditis was reported in Japan (*13*). This is probably the first case report of *B. holmesii* infection in Asia. Previous surveillance studies conducted in the United States and Canada have shown low rates (0.1%–0.3%) of *B. holmesii* infection in patients with cough (*7**,**8*). However, in a recent study, *B. holmesii* DNA was detected in 20% of NPS specimens collected from patients in France who had been given a diagnosis of *B. pertussis* infection (*14*). These surveillance data indicate that *B. holmesii* infection is present in adolescents and adults, and that the organism is associated with pertussis-like symptoms. However, other causes of viral or bacterial respiratory infection cannot be excluded. Because of lack of specific diagnostic tools to detect bordetellae, *B. holmesii* infection may have been underestimated. Accurate diagnosis and further studies are required to fully elucidate the nature of *B. holmesii* infection.
